# Circulating Pro-inflammatory Cytokines Do Not Explain Interindividual Variability in Visceral Sensitivity in Healthy Individuals

**DOI:** 10.3389/fnins.2022.876490

**Published:** 2022-07-04

**Authors:** Robert J. Pawlik, Liubov Petrakova, Lisa Brotte, Harald Engler, Sven Benson, Sigrid Elsenbruch

**Affiliations:** ^1^Department of Medical Psychology and Medical Sociology, Ruhr University Bochum, Bochum, Germany; ^2^Institute of Medical Psychology and Behavioral Immunobiology, Center for Translational Neuro- and Behavioral Sciences, University Hospital Essen, University of Duisburg-Essen, Essen, Germany; ^3^Department of Neurology, Center for Translational Neuro- and Behavioral Sciences, University Hospital Essen, University of Duisburg-Essen, Essen, Germany; ^4^Institute for Medical Education, Center for Translational Neuro- and Behavioral Sciences, University Hospital Essen, University of Duisburg-Essen, Essen, Germany

**Keywords:** visceroception, visceral sensitivity, visceral pain, gut-brain axis, cytokines, inflammation, stress, anxiety

## Abstract

A role of the immune system in the pathophysiology of pain and hyperalgesia has received growing attention, especially in the context of visceral pain and the gut-brain axis. While acute experimental inflammation can induce visceral hyperalgesia as part of sickness behavior in healthy individuals, it remains unclear if normal plasma levels of circulating pro-inflammatory cytokines contribute to interindividual variability in visceral sensitivity. We herein compiled data from a tightly screened and well-characterized sample of healthy volunteers (*N* = 98) allowing us to assess associations between visceral sensitivity and gastrointestinal symptoms, and plasma concentrations of three selected pro-inflammatory cytokines (i.e., TNF-α, IL-6, and IL-8), along with cortisol and stress-related psychological variables. For analyses, we compared subgroups created to have distinct pro-inflammatory cytokine profiles, modelling healthy individuals at putative risk or resilience, respectively, for symptoms of the gut-brain axis, and compared them with respect to rectal sensory and pain thresholds and subclinical GI symptoms. Secondly, we computed multiple regression analyses to test if circulating pro-inflammatory markers predict visceral sensitivity in the whole sample. Despite pronounced subgroup differences in pro-inflammatory cytokine and cortisol concentrations, we observed no differences in measures of visceroception. In regression analyses, cytokines did not emerge as predictors. The pain threshold was predicted by emotional state and trait variables, especially state anxiety, together explaining 10.9% of the variance. These negative results do not support the hypothesis that systemic cytokine levels contribute to normal interindividual variability in visceroception in healthy individuals. Trajectories to visceral hyperalgesia as key marker in disorders of gut-brain interactions likely involve complex interactions of biological and psychological factors in keeping with a psychosocial model. Normal variations in systemic cytokines do not appear to constitute a vulnerability factor in otherwise healthy individuals, calling for prospective studies in at risk populations.

## Introduction

A role of the immune system in the pathophysiology of pain and hyperalgesia has received growing attention (Grace et al., 2021), especially in the context of aversive interoceptive signals arising from the gastrointestinal tract (Chen et al., 2020; Kulkarni et al., 2021). Transdisciplinary scientific interest within basic and clinical research on the gut-brain axis has been driven by evidence that neuro-immune communication is relevant for elucidating mechanisms underlying normal and pathological interoception and visceral pain. Support for immune mechanisms comes from studies in clinical populations, especially disorders of gut-brain interactions such as the irritable bowel syndrome (IBS), which is characterized by chronic visceral pain and visceral hypersensitivity (Brierley and Linden, 2014; Grundy et al., 2019; Casado-Bedmar and Keita, 2020). In IBS, not only local mucosal but also peripheral immune system alterations, albeit subtle in magnitude when compared to patients with chronic inflammatory-bowel diseases and hence considered “low-grade”, have been observed (Ohman and Simrén, 2010; O’Malley et al., 2011; Burns et al., 2019). Markers of systemic inflammation, including circulating pro-inflammatory cytokines, reportedly correlate with gastrointestinal symptom severity (Dinan et al., 2008; Choghakhori et al., 2017; Gupta et al., 2017) as well as with neural processes relevant to sensory, emotional, and cognitive facets of visceral pain in IBS (Gupta et al., 2017; Norlin et al., 2021). Further, together with psychological risk factors like stress and anxiety, inflammatory responses contribute to the transition from acute to chronic symptoms in post-infections IBS (O’Malley et al., 2011; Talley, 2020).

Experimental studies in healthy individuals also support a potential role of inflammatory mediators in normal visceroception and visceral pain sensitivity, which is characterized by considerable interindividual variability and sensitive to modulation by psychological and biological factors, including stress, neuroendocrine, and immune mediators (Elsenbruch et al., 2014; Icenhour et al., 2019, 2020). Using experimental endotoxemia in healthy volunteers, we and others provided proof-of-concept evidence that acutely elevated pro-inflammatory cytokine levels are capable of inducing hypersensitivity (de Goeij et al., 2013; Karshikoff et al., 2015, 2016; Janum et al., 2016), including visceral and deep pain hypersensitivity (Benson et al., 2012, 2020; Wegner et al., 2014, 2015), and effectively enhance visceral pain-induced neural activation in the brain (Benson et al., 2015), likely as an integral component of sickness behavior. We could also recently show in a randomized-controlled trial testing hydrocortisone versus placebo on visceral sensitivity that acutely elevated cortisol resulted in increased visceral pain sensitivity in healthy volunteers (Benson et al., 2019). This is also relevant since cortisol is not only a crucial neuroendocrine stress mediator but also part of the normal, adaptive physiological response during acute inflammation where it increases in concert with immune mediators like cytokines.

Whether pro-inflammatory cytokines or cortisol levels in the systemic circulation in healthy individuals contribute to interindividual variability in interoceptive sensitivity to visceral stimuli remains elusive to this date. This lack of knowledge may not only be attributable to the challenges associated with standardized visceral sensitivity testing in larger samples. Visceral sensitivity is also highly complex, with substantial interindividual variability that is likely generated by a multitude of biological and psychological factors that are difficult to disentangle, especially in heterogeneous patient samples, but also in healthy controls. Existing work in volunteers supports that low levels of circulating cytokines under healthy conditions can in fact modulate central nervous system functioning (Salvador et al., 2021), but has not addressed measures of interoception in the context of the gut-brain axis. An earlier, small study from our group conducted in healthy women revealed that IL-6 plasma levels correlated with subclinical gastrointestinal symptoms, but were not associated with visceral pain threshold (Lacourt et al., 2014). Regarding normal interindividual variability in cortisol, we could recently show elevated serum levels at baseline and during experimental testing in a healthy group with elevated chronic stress, with an impact on rectal distension-induced urgency (Icenhour et al., 2020). Together, these initial findings call for replication and refinement in larger samples. In light of the close functional interconnections between the immune and stress systems not only in patients with chronic visceral pain but also in healthy individuals (Kiank et al., 2010; Meerveld and Johnson, 2018; Labanski et al., 2020), it appears timely and relevant to examine multiple putative predictor variables together, starting with a healthy sample as a basis for future work in clinical samples. To this end, we herein compiled data from a relatively large and well-characterized sample of healthy volunteers allowing us to assess associations between visceral sensitivity, quantified with pressure-controlled rectal distensions as a clinically-relevant experimental model, and three pro-inflammatory cytokines (i.e., IL-6, TNF-α, and IL-8) previously found relevant in the context of pain and the gut-brain axis (Hughes et al., 2013; Burns et al., 2019) along with cortisol and stress-related psychological variables. Based on our earlier work on related, yet distinct questions about the intricate interconnections between immune mechanisms, psychological risk and pain, we accomplished two complementary analyses that were both aimed at testing the overall hypothesis that greater pro-inflammatory cytokines in the systemic circulation - as a putative risk factor for hypersensitivity - is associated with enhanced visceroception: Firstly, we divided the sample into subgroups with distinctly higher and lower systemic cytokine profiles, respectively, based on a composite cytokine score, modelling healthy individuals at putative risk and resilience, respectively, and compared these subgroups with respect to visceral sensitivity and GI symptoms. Secondly, we computed multiple regression analyses to test *a priori*-identified putative predictor variables for visceral sensory and pain thresholds in the whole sample of participants.

## Methods

### Participants

For the purposes of the present analysis, we compiled data collected as part of two comprehensive research studies involving standardized visceral sensitivity testing, blood sampling, and questionnaire assessments in healthy men and women. Primary studies (one published within Koenen et al., 2021; the other unpublished), involved study-specific interventions targeting immune mechanisms relevant to visceral pain modulation (German Clinical Trials Register registration IDs: DRKS00016706 and DRKS00016994). Importantly, all assessments and measures used for analyses reported on herein were acquired using identical procedures, and were accomplished prior to study-specific interventions. Recruitment and screening procedures involved a standardized telephone screening, followed by a personal onsite visit involving clinical interview, questionnaires, and a medical physical examination including a rectal digital palpation as well as the assessment of blood and clinical chemistry parameters [i.e., complete blood cell count, C-reactive protein (CRP), coagulation factors, liver enzymes, renal parameters]. In addition to any indication of abnormal blood-derived laboratory measures, stringent exclusion criteria included age < 18 and > 50 years, body mass index (BMI) < 18 or > 30, CRP > 0.5 mg/dl, regular smoking or substance use, any known physical or mental health condition, regular medication use (except hormonal contraceptives, occasional use of over-the-counter medications). Elevated anxiety or depression scores on the Hospital Anxiety and Depression Inventory (HADS, subscales scores ≥ 8) (Herrmann-Lingen and Snaith, 2011) also led to exclusion from present analyses, as did evidence suggesting relevant gastrointestinal complaints (Lacourt et al., 2014) (details on questionnaires below). Given brain imaging within primary studies (not part of the present analyses), the usual exclusion criteria for magnetic resonance imaging (MRI) also applied, and structural brain abnormalities were ruled out by a neuroradiologist in all participants. Any evidence suggesting perianal tissue damage that would interfere with rectal balloon placement was also exclusionary. Pregnancy was ruled out using a commercially available pregnancy test on the day of the study (Biorepair GmbH, Sinsheim, Germany, sensitivity 10 mIU/ml). Work was conducted in accordance with The Declaration of Helsinki and approved by the ethics committee of the University Hospital Essen (protocol numbers 16-7237; 16-7272). All volunteers gave written informed consent and received financial compensation for participation.

### Experimental Procedures

As in all our studies involving experimental visceral pain, we applied highly-standardized procedures for visceral sensitivity testing, herein accomplished together with blood sampling and a comprehensive psychosocial questionnaire battery, together forming the dataset for the present analyses. Of note, for the initial compilation of data, participants were only considered if they met inclusion and exclusion criteria and complete data were available for all primary outcome measures. All work was carried out (prior to the pandemic) in a biomedical research setting at the University Hospital Essen, Germany. After arrival on the study day, participants were prepared for blood sampling and visceral sensitivity testing, i.e., placement of an indwelling intravenous catheter in the forearm and placement of a rectal balloon. After a short accommodation period, a blood sample was drawn, together with questionnaire assessment of state anxiety. Visceral sensitivity testing was promptly started.

### Visceral Sensitivity

For assessment of visceral sensitivity, rectal sensory and pain thresholds were assessed using a well-established rectal barostat distension procedure (Elsenbruch et al., 2007; Elsenbruch and Enck, 2015). Phasic ramp distensions were appliedsec by an inflatable rectal balloon catheter placed 5cm from the anal verge, connected with a pressure-controlled barostat system (modified Isobar 3 device, G & J Electronics, Toronto, ON, Canada). A staircase distension protocol with successive pressure increments was implemented as previously described (Koenen et al., 2017, 2021). Individual distensions (duration each 30 s), separated by pauses of complete deflation (duration each 30 s), were rated on a Likert-type scale. The threshold for first sensation was defined as the distension pressure when the rating changed from “no perception” to “certain perception”; the threshold for pain as the pressure when the rating changed from “perception of an urge to defecate” to “perception of pain.” If pain threshold was not reached at a maximal pressure of 50 mmHg, which typically occurs in a small percentage of healthy participants (Benson et al., 2019), the participant was *a priori* not included in this compiled dataset.

### Psychological Variables

Chronic perceived stress was assessed with the 12-item screening scale of the Trier Inventory of Chronic Stress (TICS) (Schulz and Schlotz, 1999; Petrowski et al., 2012). The self-assessment instrument quantifies individual experiences with chronic stressors in everyday life during the preceding 3 months, and provides a reliable global measure of subjectively perceived presence and frequency of chronic stressors. Likert-scale response options are “never” (0), “rarely” (1), “sometimes” (2), “often” (3), and “very often” (4), with a total score ranging from 0 to 48, and higher scores indicating greater overall stress burden. Note that we chose this questionnaire specifically for its applicability not only to research in clinical populations but also in healthy volunteers, the availability of norm values from healthy volunteers (mean TICS score of 13 corresponds to T score of 50 as the average score in the norming sample with a standard deviation of 10), thereby expanding on our early work on the role of chronic stress in the context of visceral pain (Icenhour et al., 2020).

Symptoms of anxiety and depression were quantified with the Hospital Anxiety and Depression Scale (HADS) (Herrmann-Lingen and Snaith, 2011). HADS provides a clinically-relevant and widely-used questionnaire suitable not only for patient groups but also to quantify subclinical symptoms in healthy populations. The HADS consists of two subscales (7 items each) quantifying anxiety (HADS_A) and depression (HADS_D) symptoms, respectively.

State anxiety was assessed at the time of blood sampling (i.e., immediately prior to visceral sensitivity testing) using the state version of the State Trait Anxiety Inventory (STAI-S). STAI-S scores range from 20-80, with higher scores indicating higher state anxiety (Laux, 1981; Spielberger, 1989). The scale is sensitive to acute psychosocial stress, reflecting both emotional (anxiety, tension) and physiological (arousal) components relevant to pain perception (Benson et al., 2019).

### Gastrointestinal Symptoms

Gastrointestinal (GI) symptoms were quantified with a standardized questionnaire that we routinely use in our group as it is applicable across different visceral pain conditions as well as in healthy volunteers, who also commonly experience minor GI symptoms, albeit less frequently or intensely than patients (Lacourt et al., 2014). A range of typical GI symptoms (i.e., diarrhea, constipation, vomiting, nausea, lower abdominal pain, upper abdominal pain, heartburn, postprandial fullness, bloating, loss of appetite) in the previous three months is assessed using a Likert-type response scale (0 = experience never, 1 = experience once or twice per month, 2 = experience once or twice per week, and 3 = experience more than twice a week). As in earlier studies (Lacourt et al., 2014; Icenhour et al., 2020), we computed the total sum score for analyses.

### Plasma Concentrations of Cytokines and Cortisol

Cytokines in the systemic circulation are not from a single source but originate from multiple peripheral organs and tissues. Thus, plasma levels reflect global peripheral cytokine production. For plasma concentrations of the pro-inflammatory cytokines TNF-α, IL-6, and IL-8, and the stress hormone cortisol, blood drawn from an intravenous catheter was collected into EDTA-coated tubes (S-Monovette, Sarstedt, Nümbrecht, Germany). Plasma samples obtained by centrifugation (2000 g, 10 min, 4°C) were stored at -80°C until analysis. Cytokine and cortisol concentrations were quantified using commercially available enzyme linked immunosorbent assays (Human Quantikine ELISA, R&D Systems, Minneapolis, MN, United States for cytokines; Cortisol ELISA, IBL International, Hamburg, Germany for cortisol) according to manufacturer instructions, and assessed on a Fluostar OPTIMA Microplate Reader (BMG Labtech, Offenbach, Germany). Assay sensitivities were 0.7 pg/ml for IL-6; 0.13 pg/mL for IL-8 (HS ELISA); 0.11 pg/ml for TNF-α (HS ELISA); 0.08 ng/ml for cortisol.

Cytokine composite scores were computed as the sum of the raw three assessed cytokines concentrations for each participant (IL-6, IL-8, and TNF-α), in line with other work in the field (Andaluz-Ojeda et al., 2012; Matsumoto et al., 2018; Samanta et al., 2018). Note that since the composite score is a sum score, missing individual cytokine data or exclusion of an individual cytokine as outlier prevent its computation or valid interpretation. We herein refrained from imputing values, and only included individuals with complete cytokine values into the compiled dataset. Based on the cytokine composite scores, quartiles were computed, and subgroups with the highest and lowest quartiles, representing individuals with high and low cytokine composite scores, respectively, were compared. For statistical analyses, all cytokine and cortisol data were log-transformed (log_10_).

### Statistical Analyses

All statistical analyses were conducted using SPSS version 27.0 (IBM Corporation, Armonk, NY, United States). Subgroups with high or low cytokine composite scores, respectively, created based on quartiles, were compared for group characteristics using independent sample t-tests or Chi-Square tests where appropriate. For main research questions on visceral sensitivity and GI complaints, analyses were accomplished using analysis of variance (ANOVA) or analysis of covariance (ANCOVA) with age, BMI and cortisol as covariates.

To analyze predictors of visceral sensitivity and GI symptoms within the whole sample, multiple regression analyses were accomplished using a stepwise approach. Variables included were cytokine composite score, cortisol, age, BMI, GI symptoms, and all psychological questionnaire scores (TICS; HADS_A, HADS_D, STAI-S). In addition, supplementary regression analyses were computed for each individual cytokine (instead of the composite score). All results are reported as mean ± standard deviation (SD) unless indicated otherwise.

## Results

### Full Sample and Cytokine Composite Score Subgroups

**FIGURE 1:**
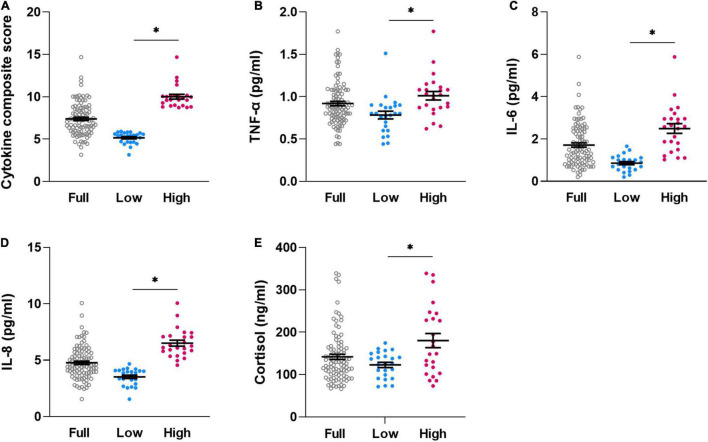
Individual dataplots for cytokine composite score **(A)**, plasma concentration of TNF-α **(B)**, IL-6 **(C)**, IL-8 **(D)**, and cortisol **(E)** for the full sample (full, *N* = 98), lowest quartile (low, *N* = 24)) and highest quartile (high, *N* = 24) subgroups based on cytokine composite score. *significant differences between subgroups, for details, see Table 1. Lines within plots indicate mean and SEM.

The compiled dataset consisted of data from *N* = 106 volunteers with complete sensitivity measures and cytokines. After exclusion of outliers for individual cytokine or cortisol concentrations (based on values > 3 standard deviations from mean), valid cytokine composite scores were computed for *N* = 98 volunteers (“full sample”; 45.0% women; for further characteristics, see Table 1, left column). For analyses aiming to compare quartile subgroups with high versus low pro-inflammatory cytokine profile based on composite scores as putative risk or resilience for disturbed interoception, respectively, the highest quartile (“High-subgroup,” *N* = 24) and the lowest quartile (“Low-subgroup,” *N* = 24) were subsequently compared (Table 1, right columns). Subgroups not only differed significantly in cytokine composite scores (Figure 1A), but also in concentrations of TNF-α (Figure 1B), IL-6 (Figure 1C), and IL-8 (Figure 1D) (all *p* < 0.005), overall confirming distinctly different pro-inflammatory cytokine profiles in subgroups. Interestingly, the High-subgroup further revealed higher cortisol concentrations when compared to the Low-subgroup (Figure 1E, *p* < 0.005), whereas no subgroup differences were observed in age [t(46) = 1.58, *p* = 0.123], the proportion of men and women [χ^2^(1,48) = 0.76, *p* = 0.383] or BMI [t(46) = 0.88, *p* = 0.386] (Table 1).

**TABLE 1:** Full sample and subgroup characteristics.

	Full sample (N = 98)	Low-subgroup (N = 24)	High-subgroup (N = 24)	*P* *
Age, years	26.13 ± 5.10	24.92 ± 3.50	27.46 ± 7.05	0.123
Proportion female,% (N)	45.0 (48)	57.1 (12)	42.9 (9)	0.383
BMI	23.23 ± 2.70	22.49 ± 2.47	23.20 ± 2.74	0.386
Plasma cortisol, ng/ml	141.23 ± 59.22	122.76 ± 30.60	180.13 ± 82.25	0.*003*
Plasma TNF-α, pg/ml	0.92 ± 0.26	0.78 ± 0.22	1.01 ± 0.25	*0.002*
Plasma IL-6, pg/ml	1.72 ± 1.01	0.86 ± 0.36	2.49 ± 1.11	*0.000*
Plasma IL-8, pg/ml	4.74 ± 1.46	3.52 ± 0.74	6.51 ± 1.27	*0.000*
Cytokine composite score	7.40 ± 1.95	5.16 ± 0.68	10.01 ± 1.37	*0.000*

Data are presented untransformed as mean ± SD. *Exact p values from independent sample t-tests or Chi-Square tests comparing subgroups in the highest versus lower quartiles, respectively, of the cytokine composite score. For individual dataplots, see Figure 1.

### Visceral Sensitivity and Gastrointestinal Symptoms in Subgroups

**FIGURE 2:**
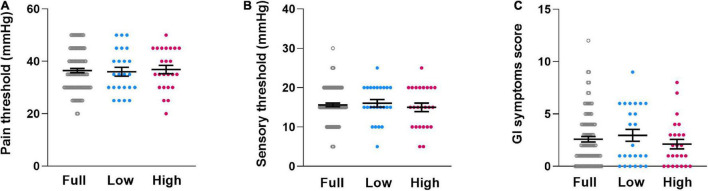
Individual dataplots for visceral pain threshold **(A)**, visceral sensory threshold **(B)** and gastrointestinal complaints **(C)** for the full sample (Full), the lowest quartile subgroup (Low) and the highest quartile subgroup (High) based on cytokine composite score. No significant subgroup differences were found. Lines within plots indicate mean and SEM.

ANOVA revealed no subgroup differences in rectal pain or sensory thresholds [pain threshold: F(1,46) = 0.126, *p* = 0.724, η_p_^2^ = 0.003, Figure 2A; sensory threshold: F(1,46) = 0.521, *p* = 0.474, η_p_^2^ = 0.011, Figure 2B]. Similarly, subgroups did not differ in reported GI symptoms [F(1,46) = 1.306, *p* = 0.259, η_p_^2^ = 0.028, Figure 2C]. Consideration of covariates (ANCOVA) did not appreciably alter results [pain threshold: F(3,44) = 0.723, *p* = 0.544, η_p_^2^ = 0.047; sensitivity threshold: F(3,44) = 1.159, *p* = 0.336, η_p_^2^ = 0.073; GI symptoms: F(3,44) = 1.949, *p* = 0.136, η_p_^2^ = 0.117].

### Psychological Variables

**FIGURE 3:**
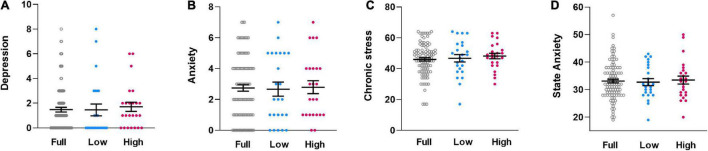
Individual dataplots for symptoms of depression **(A)**, symptoms of anxiety **(B)**, chronic stress **(C)**, and state anxiety **(D)**, assessed with validated questionnaires, for the full sample (Full), the lowest quartile subgroup (Low) and the highest quartile subgroup (High) based on cytokine composite score. No significant subgroup differences were found. Lines within plots indicate mean and SEM.

Subgroups did not differ in psychological questionnaire measures relevant to interoception and pain, specifically chronic stress [F(1,46) = 0.238, *p* = 0.628, η_p_^2^ = 0.005, Figure 3A], symptoms of depression [F(1,46) = 0.172, *p* = 0.681, η_p_^2^ = 0.004, Figure 3B], symptoms of anxiety [F(1,46) = 0.041, *p* = 0.841, η_p_^2^ = 0.001, Figure 3C]. Similarly, state anxiety assessed just prior to visceral sensitivity testing was comparable [F(1,46) = 0.169, *p* = 0.683, η_p_^2^ = 0.004, Figure 3D].

### Multiple Regression Analyses

Aiming to elucidate the putative role of pro-inflammatory cytokine levels in measures of visceroception, we computed stepwise multiple regression analyses within the full sample (Table 2). Overall, neither the cytokine composite score nor individual cytokines emerged as predictors in any of the models. As predictors of pain threshold, state anxiety (STAI-S) and chronic stress (TICS) emerged as predictors, together explaining 10.9% [F(1,95) = 5.353, *p* = 0.023, corrected R^2^ = 0.090] of the variance. In the model for sensory threshold, BMI emerged as the only predictor, explaining 5.8% variability [F(1,96) = 5.896, *p* < 0.001, corrected R^2^ = 0.048]. For GI symptoms, cortisol was the only predictor explaining 4.0% variability [F(1,96) = 4.032, *p* = 0.047, corrected R^2^ = 0.030].

**TABLE 2:** Results of multiple regression analysis (stepwise method).

Dependent variable	Predictor variable	Unstandardized coefficients		Standardized coefficients	*t*-value	*P*
		B	Std. error	B		
Pain threshold	Constant	40.472	4.386		9.227	0.000
	State anxiety (STAI-S)	-0.383	0.120	-0.335	-3.192	0.002
	Chronic stress (TICS score)	0.189	0.082	0.243	2.314	0.023
Sensory threshold	Constant	6.399	3.859		1.658	0.101
	BMI	0.401	0.165	0.241	2.428	0.017
GI symptoms	Constant	9.238	3.281		2.816	0.000
	Cortisol (log_10_)	-3.101	1.545	-0.201	-2.008	0.047

## Discussion

Aiming to shed light on the interconnections between immune mediators, psychological risk and visceroception, we analyzed data from a large, carefully screened and well-characterized sample of healthy volunteers to assess if systemic cytokine levels contribute to normal interindividual variability in interoceptive sensitivity. We compared subgroups with distinct pro-inflammatory cytokine profiles, modelling healthy individuals at putative risk or resilience, respectively, for symptoms of the gut-brain axis. Additionally, multiple regression analyses were carried out in the whole sample to assess the possible contribution of circulating pro-inflammatory cytokines, along with cortisol and psychological risk factor relevant to the pathophysiology of disorders of gut-brain interactions.

Results of both analysis approaches did not support the hypothesis that greater pro-inflammatory cytokine levels in plasma are associated with enhanced visceroception in healthy individuals. Neither did we observe differences in rectal sensitivity between two subgroups that clearly differed in pro-inflammatory cytokine composite score as well as in cortisol concentrations, nor did we find that cytokines emerged as significant predictors. These negative findings confirm and complement results of an earlier analysis carried out in a smaller healthy sample, which similarly revealed no correlation between IL-6 and visceral pain threshold (Lacourt et al., 2014). Herein, we selected the pro-inflammatory cytokines TNF-α and IL-8 in addition to IL-6, given evidence supporting increased circulating concentrations of these immune mediators in disorders of the gut-brain axis like IBS compared to healthy populations (Hughes et al., 2013; Burns et al., 2019). While several studies have provided support for altered cytokine profiles in patient samples, with higher pro-inflammatory and lower anti-inflammatory concentrations both in mucosa and blood, findings are inconsistent. However, even in studies reporting an absence of group differences in indicators of systemic inflammation, the variance is reportedly greater in patients, suggesting a role of immune activation only in a subset of patients (Bennet et al., 2016). Further, associations of cytokine levels with a range of visceral and widespread somatic symptoms have been reported irrespective of patient status (Bennet et al., 2016). Note that a direct comparison of cytokine concentrations reported herein with published data is difficult due to a number of confounding factors, including laboratory methods for cytokine measurement (e.g., choice of assay). Nevertheless, our IL-6 results observed in the High-subgroup match at least to some extent with previously published average IL-6 concentrations in IBS patients (e.g., Dinan et al., 2006; McKernan et al., 2011).

These findings are complemented by our data using experimental endotoxemia with low to moderate doses of LPS to induce acutely elevated plasma levels of pro-inflammatory cytokines in healthy individuals showing sensitization in several pain modalities and unspecific bodily sickness symptoms and psychological distress (Benson et al., 2015, 2017, 2020; Wegner et al., 2015; Lasselin et al., 2021). Of note, even though the inflammatory response observed during low dose experimental endotoxemia is considered “low grade,” circulating concentrations of pro-inflammatory cytokines are markedly higher than in our present subgroup with highest cytokine composite scores. Taking these findings together, one could speculate that there may exist a critical individual “threshold” regarding the magnitude of low-grade inflammation needed to sensitize visceral afferent signaling and/or lead to central sensitization. Moreover, more than one vulnerability factor or peripheral stress system is likely necessary to cause a detectable change in sensitivity or to induce overt GI symptoms, consistent with a bio-psycho-social model of IBS and other pain conditions associated with altered sensitivity.

Psychological factors, especially stress and anxiety, are candidates that may act alone or in concert with inflammatory mechanisms. Herein, multiple regression analyses conducted in the whole sample indeed revealed a small but significant contribution of state anxiety to rectal pain threshold. This finding replicates and complements our earlier data on mechanisms of visceroception in healthy individuals, including a correlation with state anxiety in a smaller sample (Lacourt et al., 2014), rectal hypersensitivity induced by hydrocortisone administration (Benson et al., 2019), and acute stress-induced nocebo hyperalgesia (Roderigo et al., 2017). Together, these findings are consistent with the notion that in healthy individuals, psychosocial and biological variables related to stress and anxiety constitute vulnerability factors for altered visceroception. Normal variability in circulating pro-inflammatory cytokines does not appear to play a primary role based on our findings herein, but may rather come into play as part of a vicious circle triggered by acute inflammatory challenge or more severe psychological stress. This would be in keeping with evidence that anxiety is one key psychological risk factor for the *de novo* manifestation of IBS after acute GI infection (i.e., post-infectious IBS) (Hughes et al., 2013).

In sum, our findings underscore that in healthy individuals, normal interindividual variability in interoceptive sensitivity remains difficult to explain or predict, in fact mirroring similar difficulties in patients with IBS. Further work is needed to disentangle the complex interactions between biological and psychological vulnerability factors in healthy and at-risk populations, to complement such efforts accomplished in patients with overt symptoms of IBS (Simrén et al., 2019). While we assessed a number of relevant psychological and biological factors, variability was limited by strict exclusion criteria and hence very low symptoms in our sample. Our cohort is not representative of the adult population, limiting generalizability to at risk populations or to patients with conditions of the gut-brain axis. We acknowledge the limitations arising from analyses in healthy individuals, yet view the present analyses as a first step in the sense of providing an approach towards a “healthy reference” for future work on the idea of putative risk or resilience factors related to the stress and immune systems. Furthermore, we did not assess other cytokines, chemokines, neuropeptides or neuroendocrine mediators that are closely interconnected in regulating local, peripheral and central neuro-immune communication in the context of perception and pain. Since cytokines in the systemic circulation are not from a single source but originate from multiple peripheral organs and tissues, plasma levels reflect global peripheral cytokine production. Future studies should focus on local (e.g., mucosal) inflammatory markers to complement findings herein, ideally in specific at-risk populations, such as accomplished in prospective studies on post-infectious IBS (e.g., Hughes et al., 2013). Finally, our regression results cannot indicate cause-effect relationships or identify mechanisms. Prospective studies are called for to clarify trajectories to pathology, ideally using interdisciplinary approaches at the interface of research into the gut-brain axis and psychoneuroimmunology.

## Data Availability Statement

The raw data supporting the conclusions of this article will be made available by the authors, without undue reservation.

## Ethics Statement

The studies involving human participants were reviewed and approved by Ethics committee of the University Hospital Essen, Essen, Germany. The patients/participants provided their written informed consent to participate in this study.

## Author Contributions

RJP, LP, and LB acquired data. HE, SB, and SE designed the study and acquired funding. RP, LP, and SB analyzed the data. RP, LP, SB, and SE wrote the manuscript. All authors contributed to the interpretation of the data, revised the manuscript for critical content, and approved the final version of the manuscript.

## Conflict of Interest

The authors declare that the research was conducted in the absence of any commercial or financial relationships that could be construed as a potential conflict of interest.

## Publisher’s Note

All claims expressed in this article are solely those of the authors and do not necessarily represent those of their affiliated organizations, or those of the publisher, the editors and the reviewers. Any product that may be evaluated in this article, or claim that may be made by its manufacturer, is not guaranteed or endorsed by the publisher.
